# Effects of Jae-Seng Acupuncture Treatment on the Improvement of Nasolabial Folds and Eye Wrinkles 

**DOI:** 10.1155/2015/273909

**Published:** 2015-05-04

**Authors:** Jin Hyong Cho, Ho Jin Lee, Kyu Jin Chung, Byung Chun Park, Mun Seog Chang, Seong Kyu Park

**Affiliations:** ^1^Department of Prescriptionology, College of Korean Medicine, Kyung Hee University, Seoul 130-701, Republic of Korea; ^2^O-Hang Center, Kwangdong Hospital of Traditional Korean Medicine, 612 Bongeunsa-ro, Gangnam-gu, Seoul 135-881, Republic of Korea

## Abstract

The microneedle therapy system (MTS), a mechanical method involving making minute multiple holes in the skin, reportedly improves skin condition, such as by reducing flushing and melanin. A newly attempted bloodletting therapy, Jae-Seng Acupuncture, has several advantages over traditional mechanical punching methods because it allows the practitioner to regulate the depth and direction of needle stimulations and to choose whether to stimulate the muscle layers. This study was conducted to determine the efficacy of Jae-Seng Acupuncture in the treatment of nasolabial folds and eye wrinkles. The nasolabial folds and eye wrinkles of 107 patients ranging in age from their 20s to their 70s were subjected to DermaVision, a digital skin image analyzer, before the treatment and one to six months after treatment. Additionally, stimulation of the meridians, such as Taeyang, Tongjaryo, Chongmyong, Sungup, Sabaek, Yonghyang, Chichang, Taeyong, was performed to improve the function of the stomach, large intestine. Analyses of the images indicate that Jae-Seng Acupuncture improved nasolabial folds and eye wrinkles, suggesting that this technique is a safe and effective method for the improvement of facial skin conditions.

## 1. Introduction

As the standard of living has been increasing, the demand for beauty and healthy skin has been growing within the medical area and can affect social activities. Therefore, studies on the maintenance of homeostasis and prevention of skin aging have been conducted, and alternative methods such as traditional acupuncture therapy have been explored [1].

The microneedle therapy system (MTS) is a representative approach for improving skin parameters such as wrinkles, elasticity, moisture ratio, and smoothness [2]. MTS may induce collagen synthesis by using needling to make microwounds that can stimulate fibroblast, dermal neovascularization with no change in pigmentation as observed in percutaneous collagen induction therapy [3]. Additionally, for incisional repair, platelet-derived growth factor (PDGF-BB homodimer) appears to enhance the inflammatory phase of wound healing to induce intracellular procollagen type I (PC-I) synthesis indirectly, whereas transforming growth factor-beta 1 (TGF-beta 1) enhances PC-I synthesis directly, accounting for their different durations of activities within healing wounds [4].

Building on this technique, Jae-Seng Acupuncture which means acupuncture for cell regeneration in Korean is applied by hand to make 1.0 to 3.0 mm incisions in the dermis of the skin to improve blood circulation and stimulate collagen and elastin secretion. In addition to yielding the positive effects of MTS, Jae-Seng Acupuncture is applicable to various facial regions that are difficult to access using instruments, such as the rounded side of noses and the area surrounding the eyes. Stimulating face meridians followed by Jae-Seng Acupuncture treatment improved elasticity and reduced number of pores, flushing and wrinkles of the face. This study was conducted to determine the efficacy of Jae-Seng Acupuncture in the treatment of nasolabial folds (NLs) and eye wrinkles (EWs).

## 2. Materials and Methods

Emla cream 5% (lidocaine 25 mg/g, prilocaine 25 mg/g; Astrazeneca Korea, Seoul, Republic of Korea) was used as an anesthetic. A 30 G × 1/2 needle manufactured by the International Hongchim Association connected to an 80 mm folder was used for Jae-Seng Acupuncture treatment.

### 2.1. Image Measurement and Analysis

**Figure 1:**
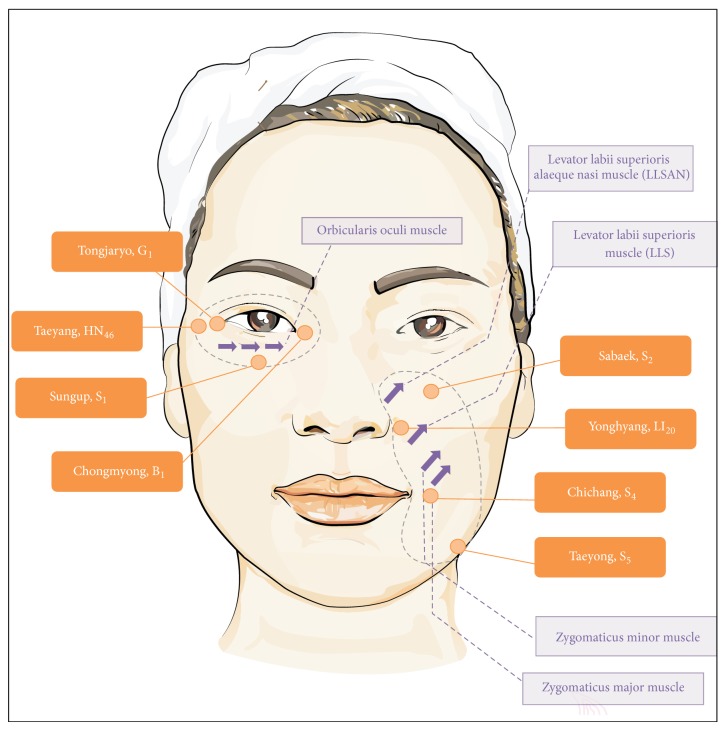
Locations of Jae-Seng Acupuncture treatment. Arrows indicate the stimulation points of the muscles in this therapy, and each meridian point is also presented. The procedure is described in Section 2.

To examine the effects of Jae-Seng Acupuncture treatment on NLs and EWs, 107 patients ranging in age from their 20s to their 70s (Table 1) were examined one to six months after the treatment. To relieve pain, the anesthetic cream was applied on the face for 30 min. DermaVision (OptoBioMed, Wonju, Korea) was used to analyze images of wrinkles, as reported previously [5, 6]. For EW treatment, the needle was injected repeatedly to the dermis at a depth of 1-2 mm in the same direction as the wrinkles to stimulate the orbicularis oculi muscle, and the temporalis muscle was stimulated by injecting the needle in a parallel direction with the skin layer at a 2 cm depth. Stimulation of the meridians such as Taeyang, Tongjaryo, Chongmyong, Sungup was performed by injecting the needle at a 2 to 3 mm depth in the vertical direction for corresponding points. For NL treatment, the needle was injected repeatedly to the dermis at a 2 to 3 mm depth in the vertical direction of the NLs, as shown in Figure 1. Next, muscles such as the levator labii superioris alaeque nasi (LLSAN), levator labii superioris (LLS), zygomaticus major, and zygomaticus minor were stimulated by injecting the needle at a 1 to 1.5 cm depth in the vertical direction. In addition, stimulation of the meridians such as Sabaek, Yonghyang, Chichang, Taeyong was performed by injecting the needle at a 1 to 1.5 cm depth in the vertical direction. For the last acupuncture session, the subcision was carried out to cut off the adhesion of wrinkles followed by calming the face with a cooling pack for 20 min. To measure results, parallel polarization images were taken by DermaVision before and after the Jae-Seng Acupuncture treatment. Figure 1 shows the facial locations where Jae-Seng Acupuncture was used.

### 2.2. Statistical Analysis

Image data were analyzed between each group using paired-sample *t*-testing for NLs and EWs. One-way analysis of variance (ANOVA) was used to analyze differences within the group before and after the Jae-Seng Acupuncture treatment. A *P* value less than 0.05 and LSD (least significant difference) were considered to indicate statistical significance.

## 3. Results

### 3.1. DermaVision Analysis

**Figure 2:**
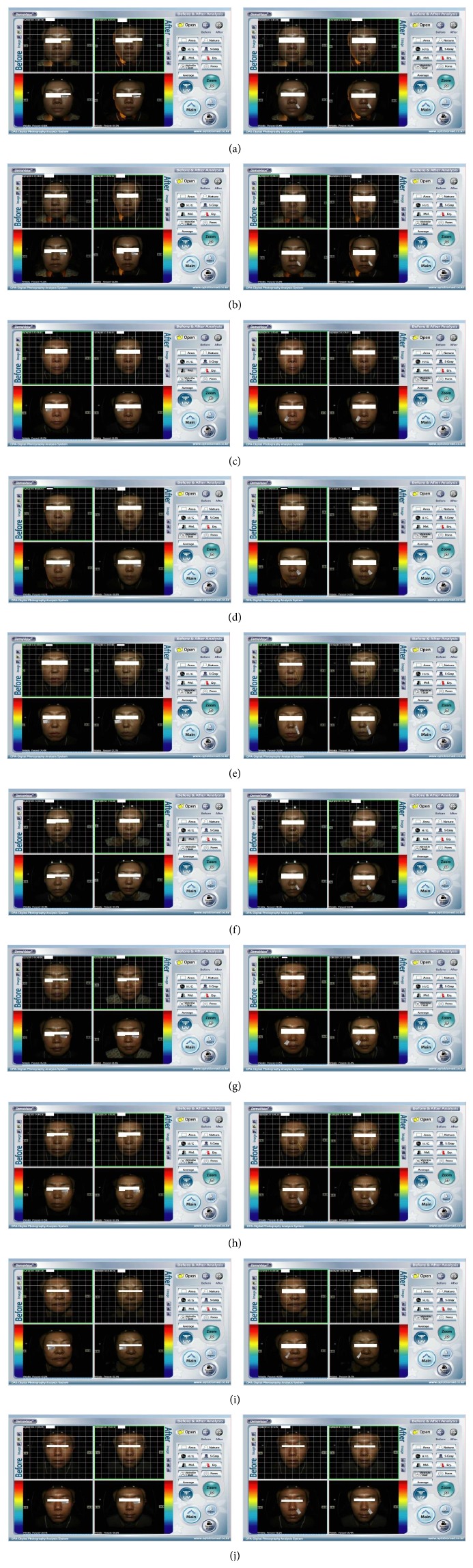
DermaVision images before and after Jae-Seng Acupuncture treatment. The results were calculated automatically as the percentage before (left) and after (right) treatment and are shown in the left panel for eye wrinkles and right panel for nasolabial folds, respectively. The figure shows the calculation, with the designated area shown as white boxes for eye wrinkles and nasolabial folds. Female, 20s (a); female, early 30s (b); female, mid-30s (c); female, late 30s (d); female, 40s (e); female, early 50s (f); female, mid-50s (g); female, 60s (h); female, 70s (i); and male, 30s (j) are shown.

DermaVision images were analyzed to compare the effects of Jae-Seng Acupuncture before and after the treatment. Results were calculated automatically as a percentage before (left) and after (right) treatment and are shown in the left panel for EWs and right panel for NLs (Figures 2(a)–2(j)). Among female patients in their 20s, EWs and NLs were improved from 42.6% to 31.5% and from 39.4% to 36.0%, respectively (Figure 2(a)). Among female patients in their early 30s, EWs and NLs were improved from 41.2% to 35.0% and from 35.9% to 32.8%, respectively (Figure 2(b)). The effects of the therapy were observed in all patient groups as follows: 40.2% to 33.9% and 41.5% to 34.0% in females in their mid-30s; 44.1% to 38.9% and 39.5% to 34.2% in females in their late 30s; 34.4% to 27.1% and 39.0% to 38.0% in females in their 40s; 38.4% to 34.1% and 38.5% to 33.4% in females in their early 50s; 45.1% to 38.9% and 34.5% to 33.4% in females in their mid-50s; 41.5% to 37.3% and 41.6% to 39.5% in females in their 60s; 42.2% to 32.1% and 40.5% to 30.7% in females in their 70s; and 38.1% to 36.5% and 39.2% to 36.4% in males in their 30s.

### 3.2. Paired-Samples *t*-Test

Right NLs before and after Jae-Seng Acupuncture treatment differed significantly (*P* < 0.001; see Table 2). The average difference before and after Jae-Seng Acupuncture treatment in right NLs was 2.63, indicating a reduction after treatment. Matching 2~4 (see Table 2) revealed similar tendencies to those observed in right NLs. Averages reductions ranged from 2.5 to 3.6, indicating improvements in NLs and EWs after Jae-Seng Acupuncture treatment.

### 3.3. Correlation Coefficient of Matching Samples

The correlation coefficient of right NLs before and after Jae-Seng Acupuncture treatment was 91.1%, indicating statistical significance at the 95% significance level (Table 3). The frequencies and correlations of changes in wrinkles ranged from 83 to 91% among the four groups, demonstrating a positive correlation after treatment.

### 3.4. Mean Difference of Treatment according to Age

**Figure 3:**
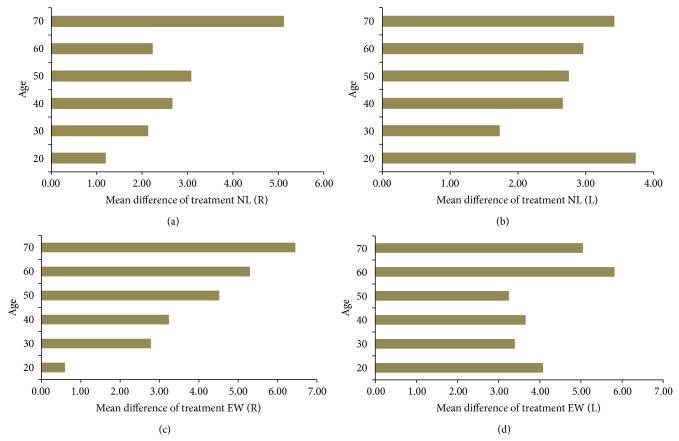
Mean difference of treatment for both nasolabial folds and eye wrinkles. The effects of the Jae-Seng Acupuncture treatment were calculated and are shown according to age. Mean difference for NL (R): (a), NL (L): (b), EW (R): (c), and EW (L): (d).

One-way ANOVA was carried out to analyze the effects of Jae-Seng Acupuncture treatment on patients of different ages. For right NLs, the mean averages according to age were in the order of 70s > 50s > 40s > 60s > 30s > 20s (Figure 3). For left NLs, the mean averages according to age were in the order of 20s > 70s > 60s > 50s > 40s > 30s. For right EWs, the mean averages according to age were in the order of 70s > 60s > 50s > 40s > 30s > 20s, while for left EWs the mean averages according to age were in the order of 20s > 70s > 60s > 50s > 40s > 30s.

## 4. Discussion

People are increasingly more interested in taking care of their appearance, and many choose to have cosmetic surgery [7]. Dermatological care is one of the most frequently used therapies to improve skin conditions including acne, scars, pigmentation, and wrinkles. Chemical and laser decortications have been widely performed, but they involve relatively long recovery times and are often accompanied by flushing, infection, and scarring [8, 9].

MTS was developed to create microscopic channels, puncturing the skin by rolling 192 needles sized 0.25~2.0 mm over the skin's surface. Automatic MTS can induce procollagen I expression and increase the weight and density of the dermis [10]. Jae-Seng Acupuncture also involves making punctures on the surface of the skin, but it involves making incisions of 0.25~2.0 mm in depth on the meridians of the face, allowing bloodletting to remove the extravasated blood, and stimulating blood circulation to activate collagen and elastin synthesis in wound healing. Incisions and bloodletting of meridian points have long been used to relieve inflammation and edema by removing abscesses and allowing blood to circulate smoothly [11, 12].

Jae-Seng Acupuncture has advantages over MTS because it is performed by hand. It can be applied to the sensitive, narrow, and thin-layer facial regions that are difficult to access using instruments. Therefore, this procedure can be extended to areas such as the rounded sides of noses and the area surrounding the eyes, with different depths to the epidermis, dermis, and muscle layer. Taken together, the results presented herein indicate that stimulation of the meridians such as Sabaek, Yonghyang, Chichang, Taeyong and Sungup to improve the function of the stomach, large intestine may affect the regeneration of skin in a positive way.

A previous study of how plastic acupuncture can improve appearance focused on improving wrinkles, melanin, flushing, and water content in 18 cases [13]. In the present study, 107 cases were analyzed before and after Jae-Seng Acupuncture treatment to examine its effects on NLs and EWs. Statistical analyses showed that Jae-Seng Acupuncture treatment improved both NLs and EWs in all of the classified age groups, and the effects lasted longer than 6 months. However, this procedure needs to be further investigated because bruises and rubefaction of the skin were frequently observed for 3 to 7 days after Jae-Seng Acupuncture treatment, and more patients in their 20s, 60s, and 70s need to be examined.

## Figures and Tables

**Table 1:** Comparison by age.

Age in years		Validity			
		Frequency	Percentage	Valid percentage	Accumulative percentage
20s		5	4.7	4.7	4.7
30s		28	26.2	26.2	30.8
40s		36	33.6	33.6	64.5
50s		28	26.2	26.2	90.7
60s		6	5.6	5.6	96.3
70s		4	3.7	3.7	100.0

	Total	107	100.0	100.0	—

**Table 2:** Paired-sample *t*-test.

	Matching difference					*t* value	D.O.F.	*P* value
	Average	S.D.	S.E.	95% C.I.				
				Lower limit	Upper limit			
Matching 1^a^	2.6355	2.5487	.2464	2.1470	3.1240	10.697	106	.000
Matching 2^b^	2.5393	2.5896	.2503	2.0429	3.0356	10.143	106	.000
Matching 3^c^	3.5673	3.0722	.2970	2.9785	4.1561	12.011	106	.000
Matching 4^d^	3.6738	3.2593	.3151	3.0491	4.2985	11.660	106	.000

^a^NLB (R) versus NLA (R);

^b^NLB (L) versus NLA (L);

^c^EWB (R) versus EWA (R);

^d^EWB (L) versus EWA (L).

NL: nasolabial fold, EW: eye wrinkle, B: before, A: after, R: right, L: left, S.D.: standard deviation, S.E.: standard error of the average, C.I.: confidence interval for the difference, and D.O.F.: degree of freedom.

**Table 3:** Matching sample correlation coefficient.

	*N*	Correlation coefficient	*P* value
Matching 1^a^	107	.911	.000
Matching 2^b^	107	.911	.000
Matching 3^c^	107	.880	.000
Matching 4^d^	107	.828	.000

^a^NLB (R) versus NLA (R);

^b^NLB (L) versus NLA (L);

^c^EWB (R) versus EWA (R);

^d^EWB (L) versus EWA (L).

NL: nasolabial fold, EW: eye wrinkle, B: before, A: after, R: right, and L: left.
